# Case Report: Continuous antegrade liposomal amphotericin B irrigation as an adjunct to endoscopic management of a refractory renal fungal bezoar

**DOI:** 10.3389/fmed.2026.1965455

**Published:** 2026-10-23

**Authors:** Dania Ghaziri, Alaa Balaghi, Ghassan Al Awar, Jad Hamawi, Maher Abdessater

**Affiliations:** 1 Department of Pharmacy, American University of Beirut Medical Center, Beirut, Lebanon; 2 Division of Urology, Department of Surgery, American University of Beirut Medical Center, Beirut, Lebanon; 3 Department of Internal Medicine, Division of Infectious Diseases, American University of Beirut Medical Center, Beirut, Lebanon

**Keywords:** Candida lusitaniae, case report, liposomal amphotericin B, nephrostomy irrigation, renal fungal bezoar

## Abstract

**Background:**

Obstructing renal fungal bezoars are rare but challenging complications of upper urinary tract candidiasis. Systemic antifungal therapy alone is often inadequate because of the high fungal burden and associated urinary obstruction. Current guidelines recommend urinary decompression, source control, systemic antifungal therapy, and adjunctive local amphotericin B deoxycholate irrigation through a nephrostomy tube when available. However, published experience with continuous antegrade liposomal amphotericin B irrigation is extremely limited.

**Case description:**

We describe a 26-year-old female patient with spina bifida, neurogenic bladder, recurrent urinary tract instrumentation, and a presumed right staghorn calculus who was found to have a large obstructing right renal fungal bezoar. Following failure of systemic fluconazole and incomplete mechanical fragmentation during retrograde ureteroscopy, continuous antegrade liposomal amphotericin B irrigation (100 mg in 500 mL of 5% dextrose at 20 mL/hour) was administered through a nephrostomy tube for 6 days. Repeat endoscopic intervention demonstrated a markedly softer and less adherent fungal bezoar, allowing uncomplicated complete removal. The patient experienced no treatment-related adverse events and recovered uneventfully.

**Conclusion:**

This case describes the use of continuous antegrade liposomal amphotericin B nephrostomy irrigation as an adjunctive treatment for refractory obstructing renal fungal bezoar. Although the independent effect of local irrigation cannot be established from a single case, the observed intraoperative change in bezoar consistency suggests a potential therapeutic benefit. Additional clinical experience and prospective studies are needed to define its optimal administration, safety, and efficacy.

## 1 Introduction

Fungal bezoars of the urinary tract are rare but potentially serious complications of upper urinary tract candidiasis. They are most commonly caused by Candida species and typically occur in patients with predisposing factors such as urinary tract obstruction, indwelling urinary catheters or stents, nephrostomy tubes, diabetes mellitus, prolonged antibiotic exposure, immunosuppression, or structural urinary tract abnormalities. Fungal bezoars may lead to persistent obstruction, hydronephrosis, recurrent infection, and acute kidney injury; and often respond poorly to systemic antifungal therapy alone because of the large fungal burden and limited drug penetration into the obstructing fungal mass (1). Consequently, source control through urinary decompression, endoscopic or surgical removal when feasible, and local antifungal instillation have been described as important adjunctive therapeutic strategies (2). Continuous antegrade amphotericin B irrigation through a nephrostomy tube represents a minimally invasive option for selected patients with obstructing fungal bezoars who are poor surgical candidates or have refractory disease. It has been described with amphotericin B deoxycholate (2); however, for liposomal amphotericin B, our literature review suggests that only one report has described continuous antegrade nephrostomy irrigation in an infant, while adult experience remains limited (3).

We present a challenging case illustrating the use of continuous antegrade liposomal amphotericin B nephrostomy irrigation in a medically complex young patient with an obstructing renal fungal bezoar.

## 2 Case description

This is the case of a 26-year-old female patient with a recent complex urological history. Her past medical history includes spina bifida, status post ventriculoperitoneal shunt insertion, neurogenic bladder managed with intermittent catheterization three times daily, epilepsy treated with valproic acid, and an atrophic left kidney with a baseline Creatinine value of 0.50–0.60 mg/dL. She initially presented in April 2026 with obstructive urosepsis associated with a right renal pelvic density presumed to represent a staghorn calculus (Figure 1). Upon presentation, her laboratory parameters showed: white blood cell count = 19,400/cu.mm (reference range: 4,000–11,000/cu.mm), C-Reactive Protein = 304.0 mg/L (reference range: 0.0–2.5 mg/L), Creatinine=2.4 mg/dL (reference range: 0.50–1.00 mg/dL). Vital signs showed a temperature of 39 **°**C and her blood pressure deteriorated reaching 91/48 mmHg**.** She required urgent right double-J ureteral stent insertion followed by right and left nephrostomy placement because of recurrent sepsis and persistent bilateral hydronephrosis (Table 1).

**Figure 1:**
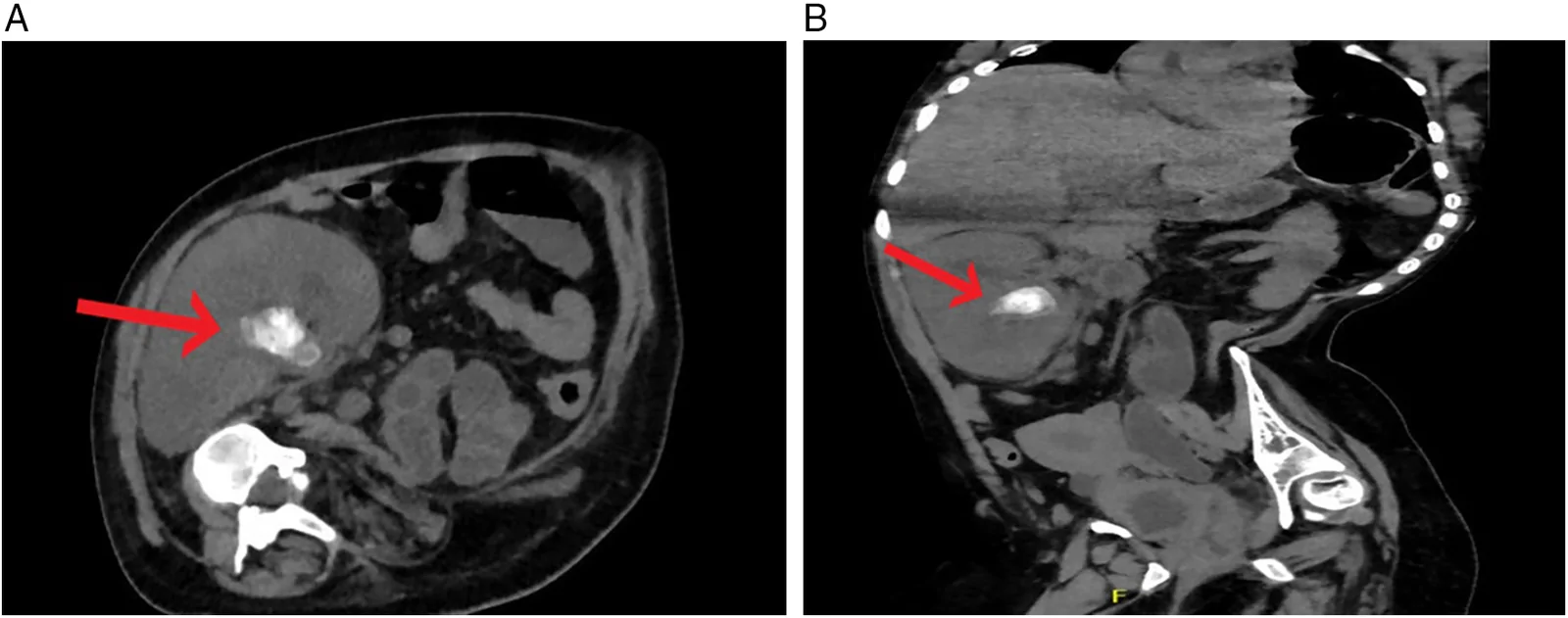
CT images of the abdomen. **(A)** Axial CT image and **(B)** sagittal CT image of the abdomen at presentation, showing a large right renal prelvic density (red arrows) initially interpreted as a staghorn calculus, with right hydronpehrosis and perinephric fat stranding. Chronic changes are also present in the left kidney. CT, computed tomography.

**Table 1:** Clinical timeline.

Date	Clinical event
2 April 2026	The patient presented with fever and developed sepsis/septic shock. Emergency right double-J ureteral stent insertion was performed for urinary decompression. She received piperacillin–tazobactam and was later discharged on ceftriaxone.
12 April 2026	The patient was readmitted with recurrent fever. Imaging suggested a presumed right staghorn calculus with worsening ascending urinary tract inflammation. A right nephrostomy tube was inserted because of persistent infection and obstruction.
20 April 2026	Left nephrostomy placed after new left hydronephrosis and drainage of purulent material; fever subsequently resolved.
24 April 2026	Right flexible ureteroscopy with attempted stone fragmentation/removal and right double-J stent exchange. A large white obstructing fungal bezoar was visualized in the right renal pelvis. Renal fungal culture grew Candida lusitaniae, and fluconazole was administered.
7 May 2026	Presented with fever and reduced left nephrostomy output. Imaging showed persistent right renal pelvic density suspicious for a fungal bezoar and a dislodged left nephrostomy tube with severe residual left hydroureteronephrosis. Meropenem, vancomycin, and fluconazole were initiated.
7 May 2026	Two left double-J stents were inserted because of the duplicated left collecting system.
8 May 2026	Treatment was adjusted to ampicillin, systemic fluconazole, and local liposomal amphotericin B administered through the right nephrostomy by continuous irrigation. Liposomal amphotericin B 100 mg in 500 mL D5W at 20 mL/hour for 6 consecutive days.
14 May 2026	Right percutaneous nephrolithotomy (PCNL) was performed. Through the percutaneous tract, the nephroscope was introduced and the fungal bezoar was completely removed using an ultrasonic LithoClast. Retrograde pyelography was performed, and a right nephrostomy tube was placed. No complications occurred.
15 May 2026	The patient was clinically and hemodynamically stable. The nephrostomy tube was clamped, with no flank pain or reduction in urine output.
16 May 2026	The patient was discharged in excellent clinical condition with a Foley catheter and follow-up plans with Urology and Infectious Diseases.

After clinical stabilization, she underwent right flexible ureteroscopy with right double-J stent exchange on 24 April 2026 for treatment of the presumed staghorn calculus. Intraoperative evaluation unexpectedly demonstrated a large white fungal bezoar occupying the right renal pelvis and causing urinary obstruction with absence of any calculus. An attempt to fragment and remove the fungal bezoar using a holmium:YAG laser at a maximum power of 20 W was unsuccessful. Fungal culture from the right renal pelvis subsequently grew Candida lusitaniae. supporting the diagnosis of an obstructing renal fungal bezoar. Systemic fluconazole 400 mg once daily was subsequently administered for two weeks. Despite systemic antifungal treatment, follow-up imaging continued to demonstrate a persistent right renal pelvic filling defect consistent with the previously identified fungal bezoar.

On 7 May 2026, the patient was readmitted with fever reaching 39.8 °C and decreased output from the left nephrostomy tube for two days. Computed tomography of the abdomen and pelvis demonstrated left nephrostomy tube dislodgement with pyelitis, persistent right renal pelvic density suspicious for the known fungal bezoar, and bilateral urinary tract inflammatory changes (Figure 2). Broad-spectrum antimicrobial therapy with meropenem, vancomycin, and fluconazole was initiated. Emergency left double-J ureteral stent insertion was performed for urinary decompression, with placement of two ureteral stents because of a duplicated left collecting system.

**Figure 2:**
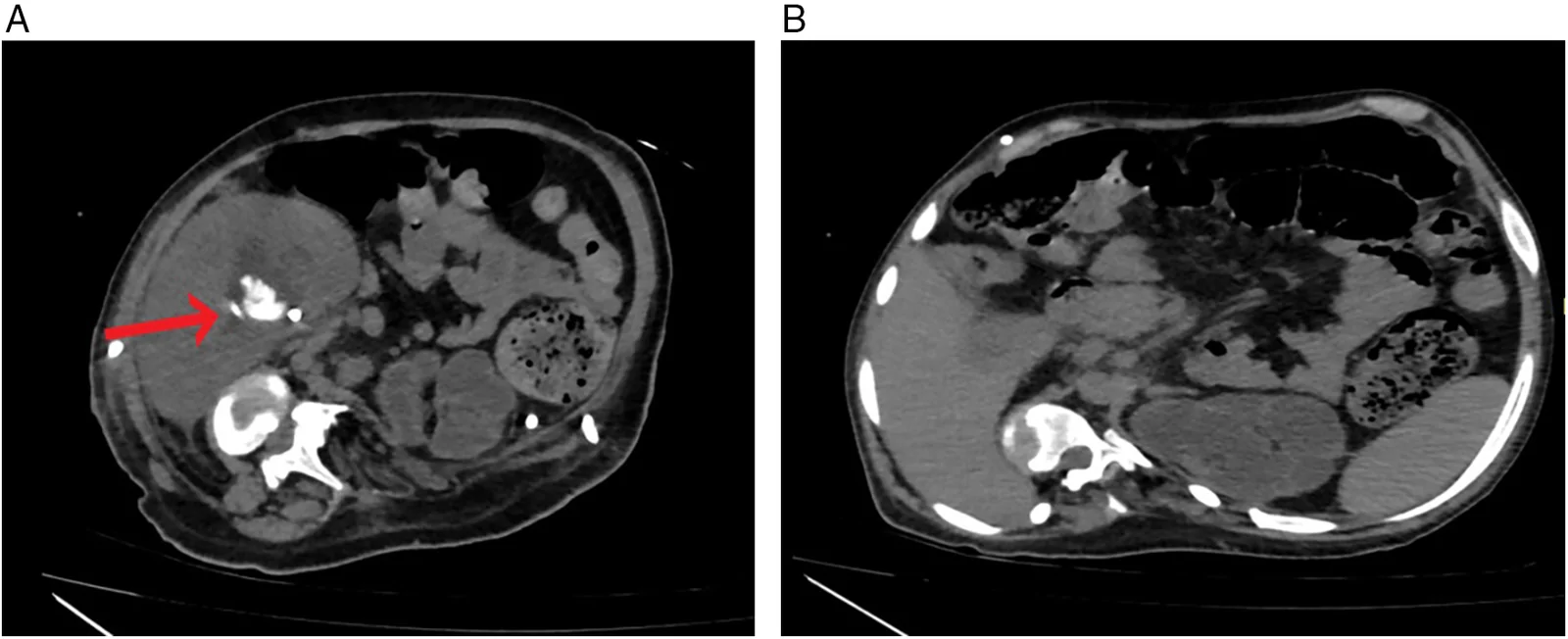
CT images of the patient. **(A and B)** Axial CT images of the abdomen and pelvis showing a persistent 2.3 × 1.5 cm right renal pelvic density (red arrow in A) consistent with the known fungal bezoar, and bilateral urinary tract inflammatory changes. CT, computed tomography.

Because the obstructing right renal fungal bezoar persisted despite systemic antifungal therapy remaining a potential source of ongoing infection and urinary obstruction, a multidisciplinary team comprising Urology, Infectious Diseases, and Clinical Pharmacy elected to administer continuous local antifungal therapy. Continuous antegrade liposomal amphotericin B irrigation was delivered through the existing right 8-Fr nephrostomy tube, which had originally been placed for persistent hydronephrosis despite ureteral stenting. Liposomal amphotericin B was prepared as 100 mg in 500 mL of 5% dextrose, corresponding to a concentration of 0.2 mg/mL, and administered continuously through the existing right nephrostomy tube at 20 mL/hour, equivalent to approximately 4 mg/hour. This corresponds to a total administered dose of approximately 550 mg. The continuous low-rate administration was selected to provide prolonged direct exposure of the fungal mass to amphotericin B while maintaining continuous drainage through the pre-existing ureteral stent and Foley catheter, as well as minimizing systemic toxicity. Treatment was continued for six consecutive days, prior to the date of the planned definitive percutaneous endoscopic intervention. Because nephrostomy administration of liposomal amphotericin B was off-label, treatment was undertaken with multidisciplinary oversight from the Urology, Infectious Diseases, and Clinical Pharmacy teams. During this irrigation period, the patient was monitored for changes in vital signs, urine output, nephrostomy-tube patency and drainage, flank pain, hematuria, and treatment-related symptoms. Renal function, serum potassium and magnesium levels, liver function, and hematologic parameters were also monitored. No treatment-related clinical abnormalities or catheter-associated complications were documented.

Following local antifungal therapy, the patient underwent right percutaneous nephrolithotomy (PCNL) on 14 May 2026. Follow-up CT demonstrated an interval reduction in size from 2.3 cm to 1.6 cm (Figure 3). Nephroscopic evaluation was performed through the established percutaneous tract, and the fungal bezoar was completely fragmented and removed using an ultrasonic probe. Retrograde pyelography confirmed satisfactory drainage, and a right nephrostomy tube was left in place. Compared with the initial ureteroscopic findings, the fungal bezoar was noted intraoperatively to be significantly softer and less adherent, facilitating complete removal without intraoperative complications (Supplementary Figure 1). The patient tolerated the procedure well, and no procedure-related complications occurred.

**Figure 3:**
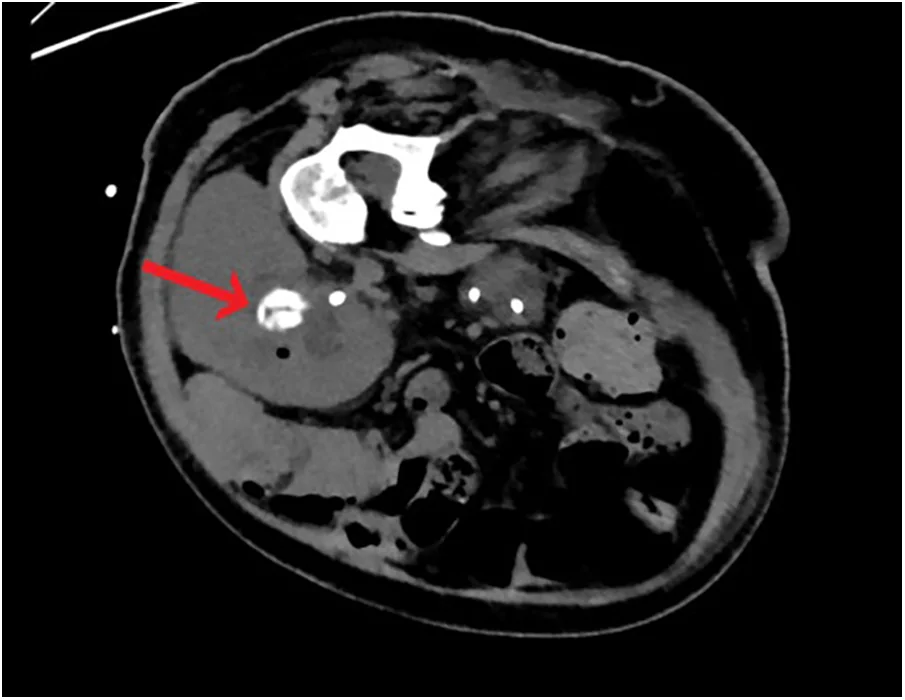
Follow-up CT obtained after local antifungal irrigation. Follow-up axial CT image of the abdomen demonstrating a persistent right renal pelvic density corresponding to the known fungal bezoar, with an interval decrease in size from 2.3 × 1.5 cm to 1.6 × 1.5 cm. The associated calyceal dilatation remained unchanged despite ureteral stenting, raising concern for possible double-J stent dysfunction. CT, computed tomography.

The postoperative course was uneventful. The nephrostomy tube was clamped on postoperative day 1 after confirmation of adequate urinary drainage and clinical improvement. Throughout her hospitalization, the patient remained afebrile, hemodynamically stable, and without flank pain or deterioration in renal function.

She was discharged in excellent clinical condition on oral fluconazole 300 mg daily with a Foley catheter *in situ*. She was provided with instructions regarding expected postoperative symptoms, including dysuria, mild hematuria, and moderate discomfort, as well as warning signs requiring urgent medical attention, such as fever, chills, or urinary retention. Follow-up with the Urology and Infectious Diseases teams was arranged within two and ten days, respectively. At the two-month follow-up, CT demonstrated no residual right renal pelvic fungal bezoar (Supplementary Figure 2) and urine cultures did not grow any organisms. Full assessment for condition-related complications was performed with no detected abnormalities.

## 3 Discussion

Fungal bezoars of the upper urinary tract are an uncommon manifestation of complicated upper urinary tract candidiasis and pose a significant therapeutic challenge as they lead to both infection and mechanical obstruction. They may mimic staghorn calculi on cross-sectional imaging by conforming to the pelvicalyceal system in a branching configuration, potentially delaying diagnosis until endoscopic evaluation (4, 5). Reported risk factors include urinary tract abnormalities, indwelling urinary devices, nephrolithiasis, prolonged antibiotic exposure, diabetes mellitus, and immunocompromising conditions (1). In this patient, neurogenic bladder requiring intermittent catheterization, bilateral urinary tract instrumentation, nephrostomy tubes, ureteral stents, recurrent urological interventions, and recurrent urinary tract obstruction likely contributed to fungal colonization and bezoar formation.

Management of urinary fungal bezoars differs substantially from that of uncomplicated candiduria. Because fungal bezoars serve as both a persistent infectious nidus and a mechanical source of urinary obstruction, systemic antifungal therapy alone is frequently inadequate. Limited penetration of antifungal agents into the densely organized fungal mass, together with impaired urinary drainage, often results in persistent infection despite microbiological susceptibility. Accordingly, the 2016 IDSA guidelines recommend urinary tract decompression, surgical or endoscopic removal when feasible, systemic antifungal therapy, and adjunctive nephrostomy irrigation with amphotericin B deoxycholate, 25–50 mg in 200–500 mL of sterile water when a nephrostomy tube is present (2). Amphotericin B is a large polyene antifungal medication existing in multiple forms, including amphotericin B deoxycholate and the later-developed lipid-based formulations, including the liposomal form. Both formulations differ in terms of carriers, mechanisms of delivery, and distribution. At the molecular level, amphotericin B deoxycholate is organized in a micellar distribution, freely binding to the fungal cell membrane through ergosterol. On the other hand, the liposomal form releases the amphotericin B molecule from the liposome once it comes in contact with the fungal cell membrane, this molecule eventually binds to ergosterol, leading to the formation of pores in this membrane (6, 7). Given the unavailability of amphotericin B deoxycholate in the country, the approach involved the liposomal form of amphotericin B. However, the 2016 ISDA does not recommend liposomal amphotericin B for local urinary irrigation or provide a dosing regimen for this formulation. Therefore, the regimen used in our patient represented an individualized, off-label strategy informed by the limited published experience (3) with direct liposomal amphotericin B irrigation and multidisciplinary assessment. It was selected following multidisciplinary review because the bezoar persisted despite urinary decompression, systemic fluconazole therapy, and unsuccessful initial endoscopic removal. Consequently, its safety, efficacy, and optimal dosing remain uncertain and require further investigation. In this case, liposomal amphotericin B was prepared as 100 mg in 500 mL of 5% dextrose (0.2 mg/mL) and infused continuously through the right nephrostomy tube at 20 mL/hour (approximately 4 mg/hour).

Published clinical experience with continuous antegrade nephrostomy irrigation remains scarce, and existing recommendations are based almost exclusively on amphotericin B deoxycholate. Evidence supporting local administration of liposomal amphotericin B is limited. To date, only one published report, by Lee et al. (3), has described continuous antegrade liposomal amphotericin B nephrostomy irrigation as part of a multimodal strategy for refractory upper urinary tract fungal infection in an infant. The present case adds an adult clinical observation regarding the feasibility of this adjunctive approach. In addition, liposomal amphotericin B has been shown to be a safer approach given the reported nephrotoxicity profile of amphotericin B deoxycholate in both adults and pediatrics populations (6, 8, 9). The evidence is reported in systemic administrations, rather than nephrostomy irrigation. Subsequently, our observation cannot establish a causal effect of liposomal amphotericin B. The patient received concurrent systemic antifungal therapy, urinary decompression, and sequential endoscopic interventions, which collectively contributed to the clinical and operative evolution. Therefore, the change in bezoar consistency should be interpreted as a temporal observation occurring after local irrigation rather than evidence of an independent treatment effect. The temporal association suggests that prolonged local exposure to liposomal amphotericin B may have contributed to altering the structural integrity of the fungal mass, thereby facilitating subsequent endoscopic extraction.

This case illustrates the feasibility of continuous antegrade liposomal amphotericin B irrigation as an adjunctive therapeutic strategy for obstructing renal fungal bezoars. After six days of continuous irrigation, repeat endoscopic evaluation demonstrated that the previously firm, cohesive, and difficult-to-fragment fungal bezoar had become noticeably softer and less adherent, allowing successful complete surgical removal without intraoperative complications. Regarding safety,he treatment was well tolerated, with no procedure-related adverse events or documented clinical evidence of amphotericin-associated toxicity.

Unlike urinary calculi, fungal bezoars are composed of fungal hyphae, inflammatory debris, necrotic tissue, and biofilm rather than crystalline mineral, resulting in endoscopic characteristics that differ substantially from those of conventional stone disease. Consequently, the choice of endoscopic approach should be individualized according to the size, location, and consistency of the fungal mass. Small, mobile fungal bezoars have been successfully managed with flexible ureteroroscopy and basket extraction, whereas larger fungal masses occupying the renal pelvis and calyceal system often require a percutaneous approach to achieve complete clearance (10–12). In our patient, the extensive pelvicalyceal involvement and dense organization of the fungal bezoar limited complete removal during the initial ureteroscopic intervention. Following continuous antegrade liposomal amphotericin B irrigation, repeat nephroscopy demonstrated a markedly softer and less adherent fungal mass, allowing definitive removal using an ultrasonic probe during PCNL. Beyond mechanical fragmentation, the ultrasonic lithotripter enabled simultaneous suction and evacuation of friable fungal debris, facilitating efficient clearance of the collecting system. A similar strategy was reported by Zhang et al. (11), who successfully treated a giant renal fungal bezoar using percutaneous nephroscopy with an ultrasonic lithotripsy system that combined fragmentation with continuous aspiration, emphasizing the importance of complete mechanical evacuation as part of multimodal therapy. Although comparative studies evaluating different endoscopic modalities are lacking, available reports suggest that an antegrade percutaneous approach may facilitate access, fragmentation, and evacuation in selected patients with large, organized pelvicalyceal fungal bezoars.

This case has several limitations. As a single-case report, it does not permit conclusions regarding the efficacy or comparative effectiveness of continuous antegrade liposomal amphotericin B irrigation. Furthermore, because systemic antifungal therapy and repeat surgical intervention were administered concurrently, the independent causal effect of local irrigation cannot be determined. However, the marked change in the consistency of the fungal bezoar observed at repeat surgery after six days of continuous irrigation is a noteworthy clinical finding that may suggest a beneficial adjunctive role for this approach. Further studies are required to evaluate its safety, optimal administration protocol, and therapeutic effectiveness in patients with obstructing urinary fungal bezoars.

In conclusion, continuous antegrade liposomal amphotericin B irrigation through a nephrostomy tube appears to be a feasible adjunctive therapeutic option for selected patients with obstructing renal fungal bezoars that are refractory to systemic antifungal therapy and challenging to manage with conventional surgical interventions alone. Although experience with this approach remains limited, our case adds an adult clinical observation concerning its potential role within a multidisciplinary management strategy. Further case reports, prospective studies, and multicenter collaborations are needed to establish the optimal formulation, dosing regimen, duration of therapy, safety profile, and clinical efficacy of local liposomal amphotericin B irrigation, as well as to determine whether it offers meaningful advantages over the currently recommended amphotericin B deoxycholate for local urinary tract administration.

## 5 Patient perspective

A patient perspective was not available for inclusion in this case report.

## Data Availability

The original contributions presented in the study are included in the article/Supplementary Material, further inquiries can be directed to the corresponding author.

## Supplementary material

SUPPLEMENTARY FIGURE S1

**Gross appearance of the extracted fungal bezoar. (A and B)** Macroscopic photographs of the fungal bezoar fragments following complete nephroscopic removal with an ultrasonic probe. A ruler is included for scale.

SUPPLEMENTARY FIGURE S2

**Follow-up CT after removal of the fungal bezoar.** Axial CT image of the abdomen obtained two months after treatment, demonstrating no residual right renal pelvic density corresponding to the previously identified fungal bezoar. Abbreviation: CT, computed tomography.
